# ^82^Se Metabolically-Labeled Yeast as a Matrix-Matched Isotope Dilution Standard for Quantification of Selenomethionine

**DOI:** 10.1021/acs.analchem.3c00152

**Published:** 2023-07-27

**Authors:** Kelly L. LeBlanc, Grégoire Hörndli, Marc-Antoine Bergeron, Zhigen Zhang, Patrick Denoncourt, Zoltán Mester

**Affiliations:** † Metrology Research Centre, National Research Council Canada, 1200 Montreal Road, Ottawa, Ontario K1A 0R6, Canada; ‡ Human Health and Therapeutics Research Centre, National Research Council Canada, 6100 Royalmount Avenue, Montréal, Québec H4P 2R2, Canada; § Lallemand Inc. 6100 Royalmount Avenue, Montréal, Québec H4P 2R2, Canada; ∥ Agriculture and Agri-Food Canada, 3600 Casavant Blvd. W., St-Hyacinthe, Québec J2S 8E3, Canada

## Abstract

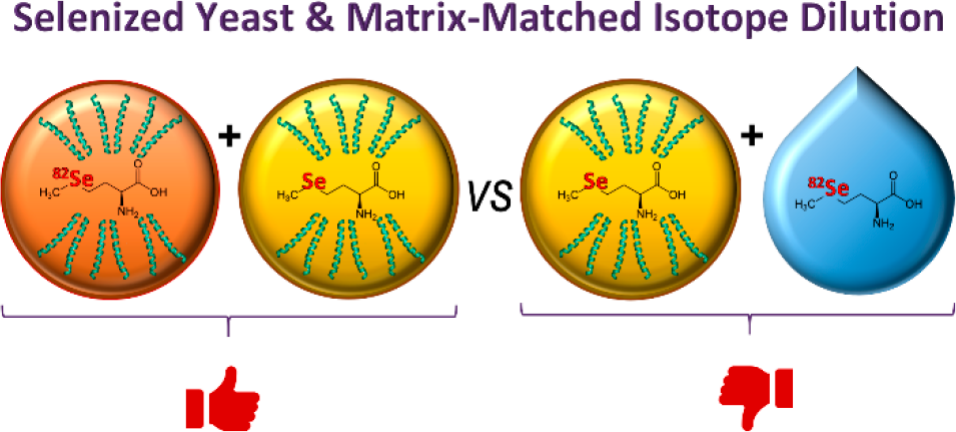

Selenized yeast is commonly used as a highly bioavailable source of selenium in dietary supplements and feed additives and is used in research settings in various disciplines due to the large number of selenium-containing metabolites formed during growth. With the selenomethionine being the major form of selenium present in selenized yeasts, its accurate quantitation is essential, however, values are frequently underestimated due to the costly and time-consuming hydrolysis-based sample preparation required to release the selenoamino acid from proteins for analysis. The National Research Council Canada has developed an 82-Se-enriched selenized yeast Certified Reference Material, SEEY-1 (DOI: 10.4224/crm.2023.seey-1) intended to be used as a matrix-matched spike material for isotope dilution analysis of selenized yeasts. The total selenium and selenomethionine contents of SEEY-1 were determined to be 322.1 ± 4.8 mg/kg (*k* = 2) and 635.6 ± 16.8 mg/kg (*k* = 2), respectively. Here we present results on the preparation of the 82-Se-enriched yeast, the certification process, and provide an example of the use of SEEY-1 as a matrix-matched spike for the analysis of selenomethionine in a sample of selenized yeast. We demonstrate here that SEEY-1 is able to compensate for the partial digestion of yeast proteins and provide reliable analytical data on Se amino acid content in under an hour instead of the 16 hours required for conventional complete acid hydrolysis.

## Introduction

Despite its history as a toxic substance, with elevated intake being associated with detrimental effects on human health,^1^ selenium (Se) is now widely recognized for its essential role in human nutrition. As selenocysteine (SeCys), the element is a vital component of 25 selenoproteins in the human proteome,^2^ which perform a variety of functions, including protection against oxidative stress and regulation of thyroid hormones.^1^

Due to the significant variations in natural levels of Se in soils worldwide, some populations experience dietary deficiencies and rely on nutritional supplements to obtain the 53–70 μg recommended for daily intake.^1,3−6^ However, the different types of Se supplements available commercially are not all equal. For example, although it is the lowest cost form of Se used in supplements, inorganic Se (selenite and selenate) is considered the least desirable because, despite its lower bioavailability, it can quickly become toxic if too large an amount is ingested. Conversely, selenomethionine (SeMet) is more bioavailable and there is a lower risk of toxicity, partially due to the fact that this species can be metabolized into selenol and selenolate species which then undergo redox cycling to generate biologically relevant selenyl (di)sulfides.^7,8^ Similarly, selenized yeast is often used in supplements, partially because 60–84% of the total Se is typically present as SeMet.^3^ In addition to this SeMet, a continuously growing list of Se-containing metabolites have been identified in selenized yeasts.^9,10^ It is likely that some of these minor species play important chemopreventative roles: a placebo-controlled study demonstrated that daily supplementation with 200 μg of Se as selenized yeast led to decreased incidences of cancer-related mortality,^11^ though a similar study supplementing with (pure) SeMet did not provide conclusive results.^12^ Based on these types of observations, researchers are continuing to investigate the biological role of SeMet and various other Se species present in selenized yeasts.^3^

Future research on the beneficial health properties of selenized yeasts depends not only on the identification of various Se-containing metabolites, but also on their accurate quantitation. As the major component in these materials, SeMet is of particular importance. Unfortunately, the fact that SeMet in yeasts is mostly found in selenium-containing proteins means its quantification is fairly labor-intensive and can pose a number of analytical challenges. The quantitative extraction of SeMet from Se-containing proteins, without unwanted degradation of the selenoamino acid, is the main issue typically faced during these analyses. Enzymatic digestions can be successful when the optimal enzyme combination is used, but quickly become very expensive when high sample throughput is required, while lower cost acid hydrolysis procedures involving HCl generally provide very low SeMet recoveries.^13^ Methanesulfonic acid reflux for sample preparation provides good recoveries while still being relatively cost-effective, but the method is still quite time-consuming, with 16 h of reflux required, and can be sensitive to variations in temperature.^14,15^

The complex sample treatment process is a significant source of uncertainty, and as a consequence, more complex calibration schemes are (often) required to mitigate these effects. For example, isotope dilution is often employed to account for variations during sample preparation, such as analyte losses or incomplete recoveries. There are numerous studies reporting the use of ^13^C- or ^i^Se-labeled SeMet isotope dilution-based quantitation of SeMet in yeast and other biological samples.^14−16^ In isotope dilution (or for that matter in any standard addition procedure) the requirement is that the (isotope) spike behaves the same way as the analyte. However, for SeMet in yeast, the SeMet is present as the free selenoamino acid in the spike, but (predominantly) as the proteinaceous form in the sample. Therefore, if SeMet is not completely lysed from the proteins during preparation,^17^ isotope dilution cannot be used to account for the incomplete analyte recovery. Conversely if the free SeMet spike breaks down or oxidizes,^18^ it would be on different time scale compared to the proteinaceous SeMet. We have attempted to address the issue of incomplete digestions/analyte decomposition by employing a small synthetic SeMet-containing peptide as calibrant instead of the free SeMet amino acid.^17^ As expected, the peptide-based SeMet standard much better mimicked the natural Se yeast during the acid digestion, yielding improved SeMet estimates. However, production of synthetic peptides at a scale required for typical food analytical chemistry is prohibitively expensive.

Based on the above considerations, the National Research Council Canada (NRC) has developed a new Certified Reference Material (CRM) for selenized yeast, called “SEEY-1”,^19^ where the yeast was enriched with isotopically labeled ^82^Se. This material is intended to serve as a matrix-matched spiking material for isotope dilution analysis.

## Experimental Section

### Safety Considerations

Methanesulfonic and nitric acids are highly corrosive and should be handled carefully, in an operational fumehood, while wearing appropriate personal protective equipment. Reflux glassware should be inspected before use to ensure its integrity. All heated samples should be allowed to cool adequately before handling.

### Certification of Total Selenium and Selenomethionine Content in SEEY-1

The Certificate of Analysis for the new matrix-matched spike material, SEEY-1, will include information relating to the mass fractions of total Se and SeMet. Details about the methods employed can be found in the Supporting Information.

### Evaluation of the Efficiency of SEEY-1 for IDMS Analysis

To confirm that SEEY-1 can be employed effectively as a matrix matched spike material for isotope dilution analysis, it was tested using NRC CRM SELM-1 (selenized yeast).^20^ Aliquots of SELM-1 were weighed into clean Teflon digestion vessels and were spiked with either SEEY-1 or NRC CRM SEES-1 (^82^Se-SeMet),^21^ in amounts to achieve approximately 1:1 ratios for ^80^SeMet:^82^SeMet. 10 mL 25% methanesulfonic acid was added, and the vessels were capped and refluxed on a hot block. One sample of each spiking method was removed after 1, 2, 5, and 16 h, then immediately filtered (0.2 μm) and stored in the refrigerator until analysis. The primary standard used for SeMet quantitation was NRC CRM SENS-1.^22^

Just prior to analysis, samples were diluted in water such that the final SeMet concentration was less than 5 mg/kg. Samples were analyzed by HPLC-ICP-QQQ-MS using an Agilent 1200 Series HPLC coupled to an Agilent 8800 Triple Quadrupole ICP-MS (Agilent Technologies, Santa Clara, California, United States). 5 μL were injected onto an Agilent Zorbax Eclipse XDB C18 column, which was held at 40 °C. A gradient elution consisting of 10 mmol/L ammonium formate at pH 5.6 and 0.1% formic acid in methanol (MeOH), at 0.4 mL/min was used: 5% MeOH from 0 to 5 min, ramping to 100% MeOH over 9 min followed by a 3 min hold, then a 0.5 min ramp back to 5% MeOH, and 6.5 min re-equilibration at 5% MeOH. (Note that to examine the water extracts, the same column and mobile phases were used, but the injection volume was 25 μL, the flow rate was 0.25 mL/min, and gradient timings were as follows: 8 min hold at 5%, 14 min ramp to 100%, 5 min hold, 1 min ramp to 5%, 10 min re-equilibration.) Due to the MeOH in the eluent, the ICP-QQQ-MS was operated in “organic mode”, utilizing a 1 mm injector, platinum cones, and the addition of a 20% O_2_ in Ar option gas. All Se isotopes (^74^Se, ^76^Se, ^77^Se, ^78^Se, ^80^Se, and ^82^Se) were monitored on-mass in triple-quadrupole mode using H_2_ as cell gas.

The concentration of SeMet was determined by isotope dilution (see the Supporting Information for details and equations). Isotope ratios (*r*_X_) were determined based on the method of Fietzke et al.,^23^ which was described with specific reference to SeMet analysis by HPLC-ICP-MS in detail in our previous work.^16^ For each ^i^Se/^82^Se ratio, the slope of the regression of the raw signal counts was determined for the points that were part of the chromatographic peak.

## Results and Discussion

The Se used for the preparation of SEEY-1 was in the form of metallic (elemental Se) pellets and, therefore, needed to be converted to a form that could be taken up by yeast during the growth process. Therefore, aliquots of the metallic Se were dissolved in 62.5% HNO_3_ in Teflon vessels, which were capped and refluxed for 1 h on a hot block set to 80 °C. The vessels were then uncapped and allowed to evaporate on the hot block until approximately 0.5 mL of solution remained, following which all aliquots were combined and diluted in DIW to achieve a final matrix of approximately 2% HNO_3_. This solution was tested via ion chromatography coupled to ICP-MS to confirm that the Se in the final solution was present as selenite. Following this analysis, 10 mol/L NaOH was added to achieve an approximate 2:1 ratio of Na:Se.

**Figure 1:**
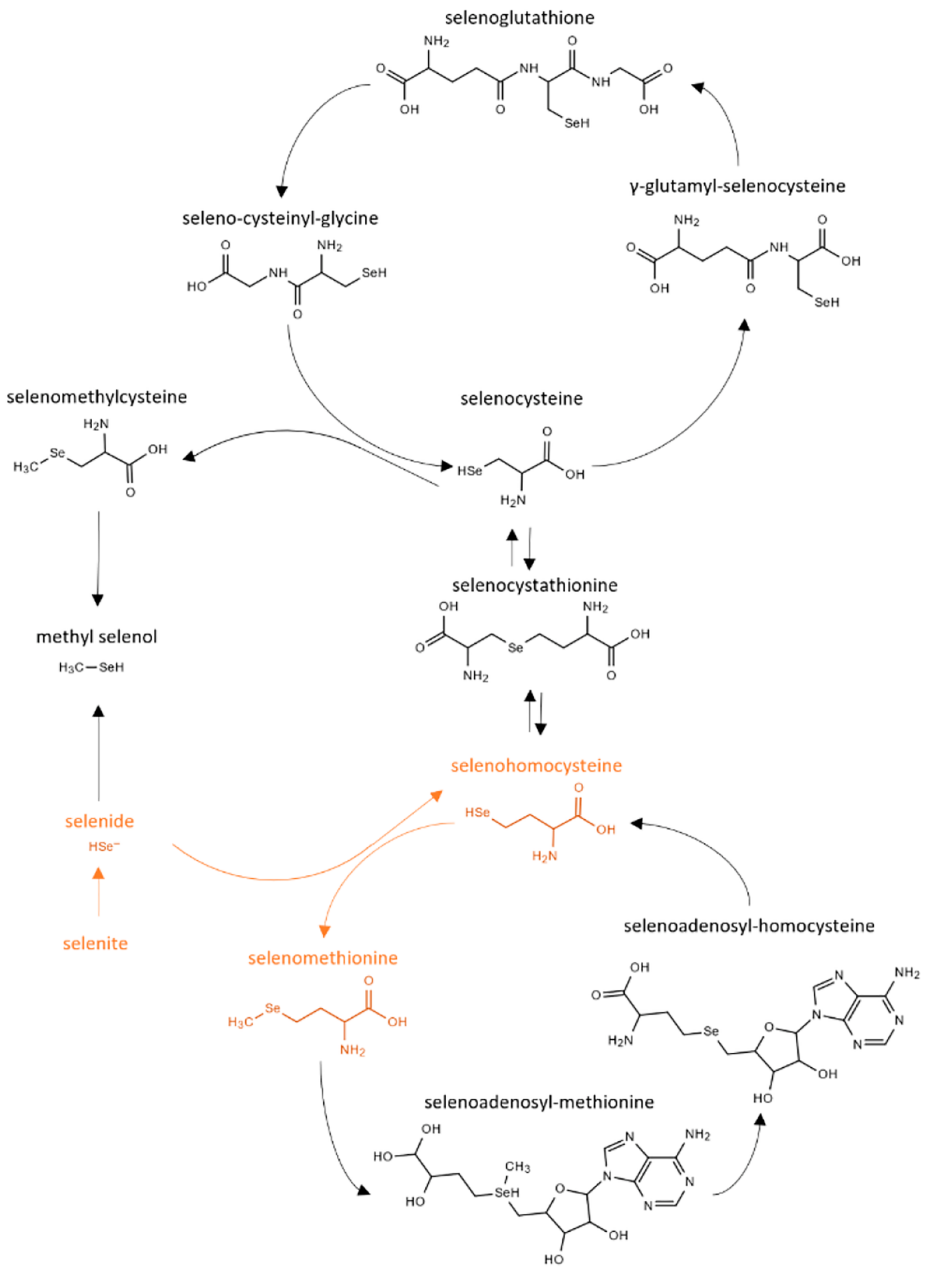
A simplified schematic of the metabolic pathway of selenium in *S. cerevisiae*, highlighting the production of selenomethionine from selenite in orange; reproduced (with modification) with permission from LeBlanc and Mester.^9^

A culture of *Saccharomyces cerevisiae* was grown in a 150 L bioreactor. Nutrients, including the ^82^Se-selenite solution were fed into the bioreactor at carefully tailored and monitored rates. A schematic outlining the metabolic pathway to arrive at SeMet from selenite is provided in Figure 1. Following culture production, the yeast was washed twice with water and spun down to a slurry of approximately 20% solids. In order to preserve sample integrity this slurry was freeze-dried (instead of heat-dried) then vacuum packed and stored in a freezer at approximately −20 °C.

The freeze-drying process resulted in large flakes of yeast, so these were transferred to a large vessel along with 1/2” and 1” Teflon balls. The vessel was rolled by hand to allow the balls to disaggregate the yeast flakes and homogenize the material. The resulting powder was sieved to remove any large particles (where possible, these were crushed and passed through the sieve). Nominal 5 g aliquots were bottled, in amber glass bottles under argon, and stored at approximately −20 °C.

### Total Selenium and Selenomethionine Content

Based on the analysis of Se primary standard NIST SRM 3149, mass biases for each ^i^Se/^82^Se ratio were determined, and these were used to correct the measured isotope ratios required for isotope dilution calculations. These mass-biased corrected isotope ratios and the known masses of each Se isotope^24^ were used to determine the atomic weight of Se in SEEY-1 to be 81.737 g/mol. Based on all of these parameters, the total Se content of SEEY-1 was determined to be 322.1 ± 4.8 mg/kg (*k* = 2).

For SeMet quantitation, isotope ratios for ^i^Se/^82^Se were determined via linear regression of the data points falling within the chromatographic peak, as described in the Experimental Section. Mass biases were determined, based on the analysis of the SeMet primary standard NRC CRM SENS-1, and used to correct the measured values. Using the mass bias corrected ratios determined for all unspiked SEEY-1 samples, the molar mass of SeMet in SEEY-1 was determined to be 198.883 g/mol and the (Se) isotopic abundance of ^82^Se-SeMet to be 0.942 (see Table 1 for abundances of other Se isotopes). Based on all these calculations, the SeMet content of SEEY-1 was determined to be 635.6 ± 16.8 mg/kg (*k* = 2), or approximately 81% of the total Se in the yeast. While both total Se and SeMet content are lower in SEEY-1 than in NRC CRM SELM-1 (2031 ± 70 mg/kg and 3190 ± 260 mg/kg, respectively, in SELM-1),^20^ the proportion of SeMet is considerably higher in SEEY-1 than in SELM-1 where SeMet accounts for 63% of the total Se content.

**Table 1:** Relative Abundance of the Isotopes of Selenium in NRC CRM SEEY-1, Based on HPLC-ICP-MS Measurements of Selenium in Selenomethionine (Showing Standard Deviation Based on Ten Measurements)

isotope	abundance in SEEY-1
^74^Se	0.001 ± 0.0001
^76^Se	0.005 ± 0.0002
^77^Se	0.004 ± 0.0002
^78^Se	0.013 ± 0.0004
^80^Se	0.035 ± 0.0007
^82^Se	0.942 ± 0.0015

A thorough uncertainty evaluation, accounting for all sources of uncertainty pertaining to the final mass fractions of total Se and SeMet in SEEY-1, was conducted to arrive at the values presented above. Similarly, experiments were performed to confirm that the material is homogeneous and stable for short-term storage or transport at various elevated temperature conditions. Details are presented in the Supporting Information.

### SEEY-1 as a Matrix-Matched Spike for IDMS

**Figure 2:**
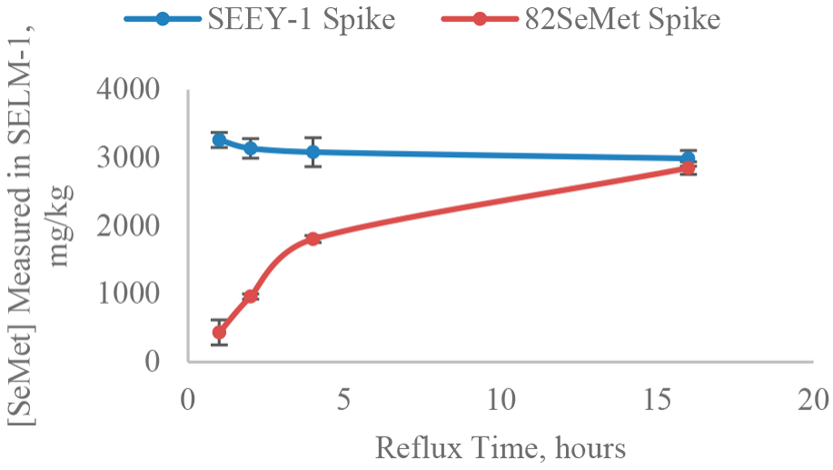
Mass fractions of SeMet measured in samples of selenized yeast SELM-1 increase over time when spiked with an ^82^SeMet chemical spike (NRC CRM SEES-1), but remain constant when spiked with ^82^Se-enriched selenized yeast (NRC CRM SEEY-1; data obtained after refluxing in 25% methanesulfonic acid for various lengths of time, where 16 h is known to achieve complete SeMet recovery from selenium-containing proteins in yeast; *n* = 3). Certified value for SeMet in SELM-1: 3190 ± 260 mg/kg (*k* = 2).

It is well established that a 16 h methanesulfonic acid reflux is effective in the complete recovery of proteinaceous SeMet from yeast.^13,25^ Therefore, when comparing results for samples of SELM-1 spiked with a matrix-match spike (SEEY-1) and a chemical spike (SEES-1), ^80^Se/^82^Se ratios at various sampling times were compared to those recorded after 16 h of reflux time to calculate the concentration of SeMet extracted from the SELM-1 sample. From the plot in Figure 2, it is apparent that this ratio (and therefore the calculated SeMet concentration) remains fairly constant, regardless of reflux time for the samples spiked with SEEY-1, but increases over time for the samples spiked with SEES-1. This is consistent with the acid hydrolysis of selenopeptides, described by McSheehy et al.^17^ Since the SeMet in SEES-1 is present as the free selenoamino acid, refluxing is not required (because it does not need to be released from a matrix) so its concentration remains constant throughout the whole sample preparation period. However, this means that if the hydrolysis of the sample is not complete, the ^80^Se/^82^Se ratio will be inconsistent and it will not be possible to correct for the low recovery. Conversely, since the SeMet in SEEY-1 is in the same form as in the yeast sample, when recovery is incomplete in the sample, it is also incomplete in the spike, and therefore isotope dilution calculations can be performed to arrive at an accurate SeMet concentration. This, of course, is based on the assumption that an adequate amount of SeMet is present in solution for detection by HPLC-ICP-MS. With an instrumental detection limit for SeMet of approximately 0.18 μg Se kg^–1^ (corresponding to about 0.1 mg Se kg^–1^ in the sample, following typical sample preparation steps and a 5-fold dilution prior to analysis), this requirement is easily met for most selenized yeast samples.

These observations are particularly important given the difficulty associated with the analysis of SeMet in yeast. Recovery is often low due to incomplete protein lysis, even when employing optimized methods for sample preparation.

As noted above, the Se content of SEEY-1 is lower than NRC’s original selenized yeast CRM, SELM-1. Ideally, an experiment would achieve a 1:1 ratio of ^i^Se:^82^Se without using a larger amount of spike material than of sample. In this scenario, ^i^Se cannot be ^74^Se or ^77^Se because they both have a lower natural abundance than ^82^Se (meaning it would be impossible to achieve the desired 1:1 ratio with ^82^Se due to its contribution in the sample). When using the three remaining isotopes for analysis, we calculated the maximum concentration of SeMet in the sample that could be analyzed without requiring a larger amount of spike than sample. For ^80^Se, this is approximately 1400 mg/kg, for ^78^Se approximately 3950 mg/kg, and for ^76^Se about 114700 mg/kg. This demonstrates that the limiting factor is at the lower end of the concentration range rather than the upper end. For samples where levels of SeMet are significantly lower, such as in dietary supplements (where selenized yeast is frequently used as the source of Se^26^), significantly smaller quantities of SEEY-1 would be required, and ^80^Se would be the ideal isotope to use for analysis.

**Figure 3:**
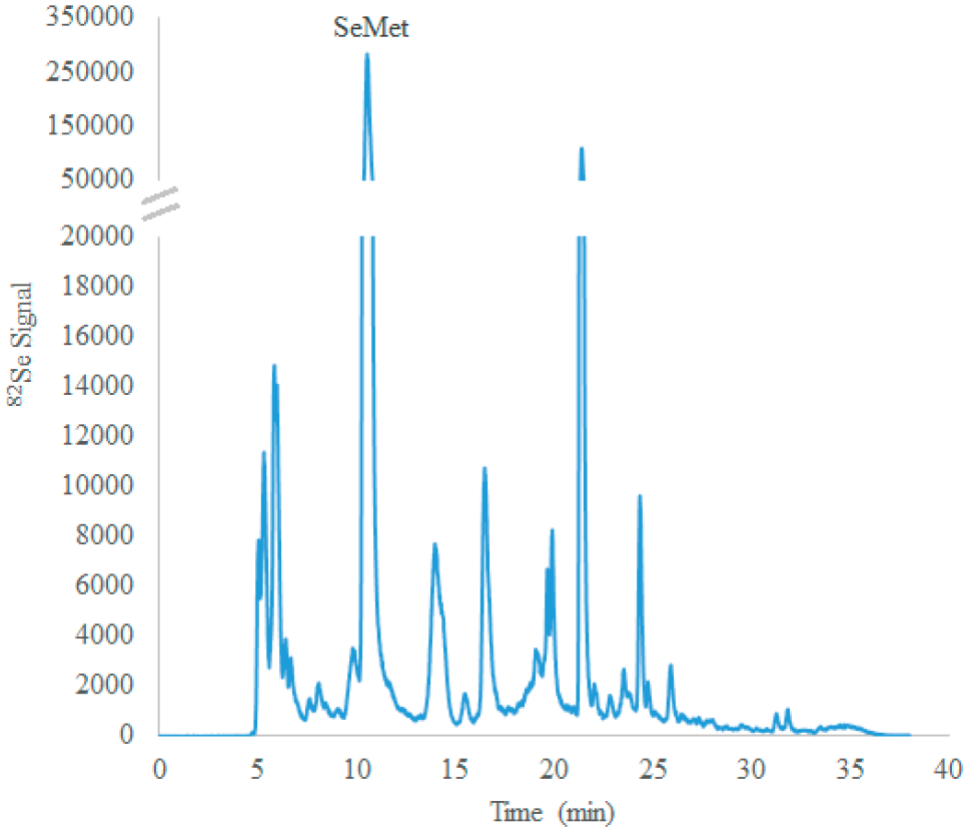
HPLC-ICP-MS chromatogram of a water extract of SEEY-1 shows 43 unique peaks, indicating a variety of Se-containing metabolites present in the yeast.

Another benefit of the use of SEEY-1 as a matrix-matched spike is related to the method of its production (growth of the yeast culture in an ^82^Se-enriched media). Because the yeast was grown naturally in the presence of selenite, SeMet, while the most abundant, is not the only isotopically labeled Se-containing metabolite present in the material. Nearly 100 unique Se-containing metabolites have already been identified in samples of selenized yeasts,^9,10^ and some preliminary analyses of aqueous extracts of SEEY-1 by HPLC-ICP-MS and HPLC coupled to high resolution MS have noted the presence of a variety of Se-containing metabolites. For example, HPLC-ICP-MS analysis of a water extract of SEEY-1 resulted in 43 unique chromatographic peaks (including SeMet) observed in the ^82^Se trace (Figure 3). When the same extract was examined following the same HPLC procedure coupled to high resolution molecular MS, exact mass searching based on our database of Se-containing metabolites^9,10^ tentatively identified 35 species (including SeMet) within 5 ppm mass error of the expected exact mass of the ^82^Se species. Some examples of tentatively identified compounds include seleno-S-adenosyl-homocysteine, seleno-biotin-sulfoxide, and the S–Se conjugate of glutathione and γ-glutamoyl-selenocysteine (or the Se–S conjugate of selenoglutathione and γ-glutamoyl-cysteine). However, without additional isotopes available in adequately high abundance for confirmation, additional work must be done to confirm the identity of these species based on their unique fragmentation patterns and, ideally, chromatographic retention time matching with a standard. Future work with SEEY-1 will aim to conclusively identify and quantify some of the metabolites present in higher relative abundances or which are known to play important biological roles. Ideally, this will extend the usability of SEEY-1 beyond the analysis of SeMet and total Se in yeasts, to the analysis of other important Se metabolites.

The structural similarities between many Se-containing metabolites produced by yeasts means that these metabolites are often categorized based on their structural features. For example, Rao et al.^27^ have suggested groupings of free selenols, oxidized selenols, and protein-bound selenols, due to the functional differences between these groups of molecules. Since analytical protocols are often developed based on these types of functional groupings, the gathering of information such as the content of total selenols (as well as the proportion in each above-mentioned category) will likely prove to be beneficial for the future use of SEEY-1.

## Conclusions

The National Research Council Canada has prepared a new Certified Reference Material, SEEY-1, which will act as a matrix-matched spike for isotope dilution analysis of total Se and Se-containing metabolites, particularly SeMet, in selenized yeasts. The use of this material will address the significant challenges associated with the rigorous sample preparation required for the analysis of proteinaceous SeMet, where values are frequently underestimated due to incomplete protein hydrolysis. Metabolically labeled “natural” proteinaceous reference materials could significantly improve the quality of measurement data and shorten analysis time required for the analysis of other amino acids as well.

## Supporting Information Available

This file contains additional details on the certification of selenomethionine and total selenium content in SEEY-1 (PDF)

## References

[ref1] Rayman M. P. Selenium and human health. Lancet 2012, 379 (9822), 1256–1268. 10.1016/S0140-6736(11)61452-9. 22381456

[ref2] Kryukov G. V.; Castellano S.; Novoselov S. V.; Lobanov A. V.; Zehtab O.; Guigó R.; Gladyshev V. N. Characterization of mammalian selenoproteomes. Science 2003, 300 (5624), 1439–1443. 10.1126/science.1083516. 12775843

[ref3] Rayman M. P. The use of high-selenium yeast to raise selenium status: How does it measure up?. Br. J. Nutr. 2004, 92 (4), 557–573. 10.1079/BJN20041251. 15522125

[ref4] Health Canada Multi-Vitamin/Mineral Supplements Monograph. http://webprod.hc-sc.gc.ca/nhpid-bdipsn/atReq.do?atid=multi_vitmin_suppl (accessed 13 May 2020).

[ref5] Institute of Medicine, Dietary Reference Intakes: Vitamin C, Vitamin E, Selenium, and Carotenoids. Panel on Dietary Antioxidants and Related Compounds; Subcommittees on Upper Reference Levels of Nutrients and Interpretation and Uses of Dietary Reference Intakes; Standing Committee on the Scientific Evaluation of Dietary Reference Intakes, National Academy Press: Washington, D.C., 2000.

[ref6] Scientific Opinion on Dietary Reference Values for selenium. EFSA Journal 2014, 12, 3846 10.2903/j.efsa.2014.3846. PMC13136995 42087966

[ref7] Rayman M. P.; Winther K. H.; Pastor-Barriuso R.; Cold F.; Thvilum M.; Stranges S.; Guallar E.; Cold S. Effect of long-term selenium supplementation on mortality: Results from a multiple-dose, randomised controlled trial. Free Radical Bio. Med. 2018, 127, 46–54. 10.1016/j.freeradbiomed.2018.02.015. 29454039

[ref8] Rayman M. P. Selenium intake, status, and health: a complex relationship. Hormones 2020, 19 (1), 9–14. 10.1007/s42000-019-00125-5. 31388899 PMC7033057

[ref9] LeBlanc K. L.; Mester Z. Compilation of selenium metabolite data in selenized yeasts. Metallomics 2021, 13 (6), na 10.1093/mtomcs/mfab031. 34156080

[ref10] LeBlanc K. L.; Mester Z. Catalogue of selenium metabolites in selenized yeast. National Research Council Canada 2020, 10.4224/40001921.

[ref11] Clark L. C.; Combs G. F. Jr.; Turnbull B. W.; Slate E. H.; Chalker D. K.; Chow J.; Davis L. S.; Glover R. A.; Graham G. F.; Gross E. G.; Krongrad A.; Lesher J. L. Jr.; Park H. K.; Sanders B. B. Jr.; Smith C. L.; Taylor J. R. Effects of Selenium Supplementation for Cancer Prevention in Patients With Carcinoma of the Skin, A Randomized Controlled Trial. J. Am. Med. Assoc. 1996, 276, 1957–1963. 10.1001/jama.1996.03540240035027. 8971064

[ref12] Lippman S. M.; et al. Effect of Selenium and Vitamin E on Risk of Prostate Cancer and Other Cancers. The Selenium and Vitamin E Cancer Prevention Trial (SELECT). J. Am. Med. Assoc. 2009, 301, 39–51. 10.1001/jama.2008.864. PMC3682779 19066370

[ref13] Yang L.; Sturgeon R. E.; McSheehy S.; Mester Z. Comparison of extraction methods for quantitation of methionine and selenomethionine in yeast by species specific isotope dilution gas chromatography-mass spectrometry. J. Chromatogr. A 2004, 1055 (1–2), 177–184. 10.1016/j.chroma.2004.09.018. 15560494

[ref14] Yang L.; Mester Z.; Sturgeon R. E. Determination of methionine and selenomethionine in yeast by species-specific isotope dilution GC/MS. Anal. Chem. 2004, 76 (17), 5149–5156. 10.1021/ac049475p. 15373455

[ref15] McSheehy S.; Yang L.; Sturgeon R.; Mester Z. Determination of methionine and selenomethionine in selenium-enriched yeast by species-specific isotope dilution with liquid chromatography-mass spectrometry and inductively coupled plasma mass spectrometry detection. Anal. Chem. 2005, 77 (1), 344–349. 10.1021/ac048637e. 15623314

[ref16] LeBlanc K. L.; Le P. M.; Meija J.; Ding J.; Melanson J.; Mester Z. Preparation and certification of natural and ^82^Se-labelled selenomethionine reference materials. J. Anal. At. Spectrom. 2021, 36, 416–428. 10.1039/D0JA00411A.

[ref17] McSheehy S.; Yang L.; Mester Z. Selenomethionine extraction from selenized yeast: An LC-MS study of the acid hydrolysis of a synthetic selenopeptide. Microchim. Acta 2006, 155 (3–4), 373–377. 10.1007/s00604-006-0520-2.

[ref18] LeBlanc K. L.; Ruzicka J.; Wallschläger D. Identification of trace levels of selenomethionine and related organic selenium species in high-ionic-strength waters. Anal. Bioanal. Chem. 2016, 408 (4), 1033–1042. 10.1007/s00216-015-9124-1. 26547190

[ref19] LeBlanc K. L.; Horndli G.; Meija J.; Grinberg P.; Beauchemin M.; Bergeron M.-A.; D’Amours D.; Dachon A.; Lock P.; Sotomey C.; Mester Z. SEEY-1: Selenium-82 labelled selenium enriched yeast Certified Reference Material. National Research Council Canada 2023, 10.4224/crm.2023.seey-1.

[ref20] Mester Z.; Brophy C.; McCooeye M.; Clancy V.; Maxwell P.; McSheehy S.; Sturgeon R.; Willie S.; Yang L. SELM-1: Selenium enriched yeast certified reference material. National Research Council Canada 2010, 10.4224/crm.2010.selm-1.

[ref21] LeBlanc K. L.; Ding J.; Meija J.; Grinberg P.; Mester Z. SEES-1: Certified Reference Material of selenium-82 labelled selenomethionine in solution. National Research Council Canada 2020, 10.4224/crm.2020.sees-1.

[ref22] LeBlanc K. L.; Le P. M.; Meija J.; Grinberg P.; Melanson J.; Mester Z. SENS-1: Certified Reference Material of natural selenomethionine. National Research Council Canada 2020, 10.4224/crm.2020.sens-1.

[ref23] Fietzke J.; Liebetrau V.; Günther D.; Gürs K.; Hametner K.; Zumholz K.; Hansteen T. H.; Eisenhauer A. An alternative data acquisition and evaluation strategy for improved isotope ratio precision using LA-MC-ICP-MS applied to stable and radiogenic strontium isotopes in carbonates. J. Anal. At. Spectrom. 2008, 23, 955–961. 10.1039/b717706b.

[ref24] IUPAC Commission on Isotopic Abundances and Atomic Weights. https://ciaaw.org/ (accessed 21 July 2022).

[ref25] Mester Z.; Willie S.; Yang L.; Sturgeon R.; Caruso J. A.; Fernández M. L.; Fodor P.; Goldschmidt R. J.; Goenaga-Infante H.; Lobinski R.; Maxwell P.; McSheehy S.; Polatajko A.; Sadi B. B. M.; Sanz-Medel A.; Scriver C.; Szpunar J.; Wahlen R.; Wolf W. Certification of a new selenized yeast reference material (SELM-1) for methionine, selenomethinone and total selenium content and its use in an intercomparison exercise for quantifying these analytes. Anal. Bioanal. Chem. 2006, 385 (1), 168–180. 10.1007/s00216-006-0338-0. 16596401

[ref26] LeBlanc K. L.; Kawamoto M. S.; Le P. M.; Grinberg P.; Nadeau K.; Yang L.; Nogueira A. R. D. A.; Mester Z. Quantitation of Selenomethionine in Multivitamins and Selenium Supplements by High Performance Liquid Chromatography Inductively-Coupled Plasma Mass Spectrometry. Food Anal. Method. 2019, 12 (6), 1316–1326. 10.1007/s12161-019-01442-6.

[ref27] Rao Y.; McCooeye M.; Windust A.; Bramanti E.; D’Ulivo A.; Mester Z. Mapping of selenium metabolic pathway in yeast by liquid chromatography-orbitrap mass spectrometry. Anal. Chem. 2010, 82 (19), 8121–8130. 10.1021/ac1011798. 20825195

