# Supplementary material for: 82Se Metabolically-Labeled Yeast as a Matrix-Matched Isotope Dilution Standard for Quantification of Selenomethionine
Source: Anal Chem. 2023 Jul 27;95(31):11583–8. doi: 10.1021/acs.analchem.3c00152 (PMC10413320; doi:10.1021/acs.analchem.3c00152)
Supplement: Supplementary file 1 — ac3c00152_si_001.pdf [file ac3c00152_si_001.pdf]

Supplementary Information for:

^{82}Se Metabolically-Labelled Yeast as a Matrix-Matched Isotope Dilution Standard for Quantification of Selenomethionine

Kelly L. LeBlanc[1]*, Grégoire Hörndli[2], Marc-Antoine Bergeron[2], Zhigen Zhang[3], Patrick Denoncourt[4], Zoltán Mester[1]

[1] Metrology Research Centre, National Research Council Canada, 1200 Montreal Road, Ottawa, Ontario, Canada

[2] Human Health and Therapeutics Research Centre, National Research Council Canada, 6100 Royalmount Ave, Montréal, Québec, Canada

[3] Lallemand Inc. 6100 Royalmount Ave, Montréal, Québec, Canada

[4] Agriculture and Agri-Food Canada, 3600 Casavant Blvd. W., St-Hyacinthe, Québec, Canada

* Kelly.LeBlanc@nrc-cnrc.gc.ca

<u>Contents of the Supplementary Information Section</u>:

Additional Details on the Certification of Selenomethionine and Total Selenium Content in SEEY-1

Page S2 Method for Total Selenium in SEEY-1

Page S3 Method for Selenomethionine in SEEY-1

Homogeneity and Stability

Uncertainty Evaluation

Page S4 Homogeneity and Stability Results

Page S5 References for the Supplementary Information

Additional Details on the Certification of Selenomethionine and Total Selenium Content in SEEY-1

Method for Total Selenium in SEEY-1

250 mg aliquots of SEEY-1 were weighed into pre-cleaned Teflon microwave digested vessels. Aliquots of a primary standard solution, prepared from diluted NIST SRM 3149 (selenium standard solution; National Institute of Standards and Technology, Gaithersburg, Maryland, United States), were added to each vessel such that the ^{80}Se:^{82}Se was approximately 1:1. One SEEY-1 sample was prepared without a primary standard spike, and one sample of NRC CRM SELM-1 (selenized yeast) spiked with an appropriate volume of ^{82}Se solution (~ 200 mg/kg) was carried as quality control. 7 mL HNO_3 and 0.5 mL 30% H_2O_2 were added to each vessel, which were capped and digested in an Anton Paar Multiwave 3000 Microwave (Graz, Austria) by ramping the power to 1400 W over 15 min, holding there for 30 min, then cooling at 0 W. Once cooled, samples were transferred to HDPE bottles and diluted with DIW to a final matrix of ~2% HNO_3. Additionally, three blends of NIST SRM 3149 and ^{82}Se solution were prepared in 2% HNO_3, such that the total Se concentration was similar to that in the samples and one blend had an ^{80}Se:^{82}Se ratio of approximately 1:1.

Analysis of total Se was performed on an Agilent 8800 triple quadrupole ICP-MS (ICP-QQQ-MS; Agilent Technologies, Santa Clara, California, United States). O_2 was used as a cell gas to monitor the Se^+ > SeO^+ transition for all Se isotopes (^{74}Se, ^{76}Se, ^{77}Se, ^{78}Se, ^{80}Se, and ^{82}Se). Quantitation of the Se content was carried out using isotope dilution based on Equation 1 for SELM-1 or Equation 2 for SEEY-1:

$$w_A = w_B \cdot \frac{r_B - r_{AB}}{r_{AB} - r_A} \cdot \frac{m_{B(AB)}}{m_{A(AB)}} \cdot \frac{\sum R_A}{\sum R_B} \cdot \frac{M_A}{M_B} \quad (1)$$

and

$$w_B = w_{A^*} \cdot \frac{r_{A^*B} - r_{A^*}}{r_B - r_{A^*B}} \cdot \frac{m_{A^*}}{m_B} \cdot \frac{\sum R_B}{\sum R_{A^*}} \cdot \frac{M_B}{M_{A^*}} \quad (2)$$

where:

A analyte in sample SELM-1

A^* analyte in primary standard (NIST SRM 3149, natural isotopic composition)

B analyte in isotopically enriched standard or sample (SEES-1 or SEEY-1)

AB mixture of sample and enriched standard

A^*B mixture of primary standard and enriched standard or sample

M_X molar mass of X ($X = A$, A^*, or B)

w_X mass fraction of X ($X = A$, A^*, or B)

$m_{X(XY)}$ mass of X used to prepare the blend of X and Y ($X,Y = A$, A^*, or B)

R_X isotope ratio in X ($X = A$, A^*, or B)

r_X isotope ratio in X as measured by mass spectrometry ($X = A$, A^*, or B)

Method for Selenomethionine in SEEY-1

Samples were prepared for SeMet analysis following a method modified from Yang *et al.*[1, 2] Briefly, 250 mg of SEEY-1 were weighted into an Erlenmeyer flask fitted with a ground glass joint. 24 mL 25% methanesulfonic acid and some clean glass beads were added to the flask which was placed on a hot plate and fitted to a water-cooled condenser. The solution was refluxed for 16 hours, then cooled, filtered (0.2 µm), and stored at approximately 4°C until analysis. Every SEEY-1 sample was run in duplicate, with one replicate being spiked, prior to refluxing, with a volume of a solution of NRC CRM SENS-1 (natural SeMet) required to obtain a 1:1 ratio for ^{80}SeMet:^{82}SeMet. In addition to SEEY-1 samples, SELM-1 was analyzed as a quality control, with replicates being spiked with NRC CRM SEES-1 (^{82}Se-selenomethionine) for isotope dilution. Additionally, three blends of SENS-1 and SEES-1 were prepared and subjected to the same reflux procedure.

Just prior to analysis, samples were diluted in DIW such that the final SeMet concentration was less than 5 mg/kg. Samples were analyzed by HPLC-ICP-QQQ-MS using an Agilent 1200 Series HPLC coupled to an Agilent 8800 Triple Quadrupole ICP-MS (Agilent Technologies, Santa Clara, California, United States). 5 µL were injected onto an Agilent Zorbax Eclipse XDB C18 column which was held at 40°C. A gradient elution consisting of 10 mmol/L ammonium formate at pH 5.6 and 0.1% formic acid in methanol (MeOH), at 0.4 mL/min was used: 5% MeOH from 0-5 min, ramping to 100% MeOH over 9 min followed by a 3 min hold, then a 0.5 min ramp back to 5% MeOH, and 6.5 min re-equilibration at 5% MeOH. Due to the MeOH in the eluent, the ICP-QQQ-MS was operated in “organic mode”, utilizing a 1 mm injector, platinum cones, and the addition of a 20% O_2 in Ar option gas. All Se isotopes (^{74}Se, ^{76}Se, ^{77}Se, ^{78}Se, ^{80}Se, and ^{82}Se) were monitored on-mass in triple-quadrupole mode using H_2 as cell gas.

The concentration of SeMet in SEEY-1 was determined following Equation 2. Isotope ratios (r_X) were determined based on the method of Fietzke *et al.*,[3] which was described with specific reference to SeMet analysis by HPLC-ICP-MS in detail in our previous work.[4] For each ^{i}Se/^{82}Se ratio, the slope of the regression of the raw signal counts was determined for the points which were part of the chromatographic peak (defined as signal-to-background greater than 250).

Homogeneity and Stability

Homogeneity was assessed through the analysis of SeMet content in eight bottles throughout the series. An isochronous stability study was performed by removing selected bottles from their storage conditions (-20°C) and placing them in controlled temperature environments (20°C and 40°C). Bottles were left in these environments for one, three, or eight weeks, then returned to the freezer such that they could be analyzed simultaneously.

Uncertainty Evaluation

For the uncertainty evaluation for both total Se and SeMet content in SEEY-1, a Monte Carlo simulation was performed (using the NIST Uncertainty Machine[5]) based on the standard uncertainties associated with each component in Equation 2, with the exception of w_A (where the uncertainty was set to 0.0001 mg/kg) as the uncertainty associated with the primary standard should be considered separately. For this calculation, uncertainties on atomic masses and isotope ratios were obtained from IUPAC,[6] and measured uncertainties in isotope ratios based on either relative standard deviation of instrument counts (for total Se) or the uncertainty of the linear regression (for SeMet). Based on the output from this computation, the relative

standard uncertainty was applied to all measured values for mass fractions of SeMet and total Se in SEEY-1. These values and associated uncertainties were input into the NIST Consensus Builder,[7] using Gaussian fitting of the Bayesian model, and the output was used to calculated the combined standard uncertainty. Measurement uncertainty was considerd as the combination of the uncertainty from the primary standard and the standard uncertainty output from the Bayesian model. The dark uncertainty component represents the uncertainty due to (in)homogeneity. Overall, the combined expanded (k = 2) relative uncertainties to SeMet and total Se in SEEY-1 were determined to be 2.6% and 1.5%, respectively.

Homogeneity and Stability Results

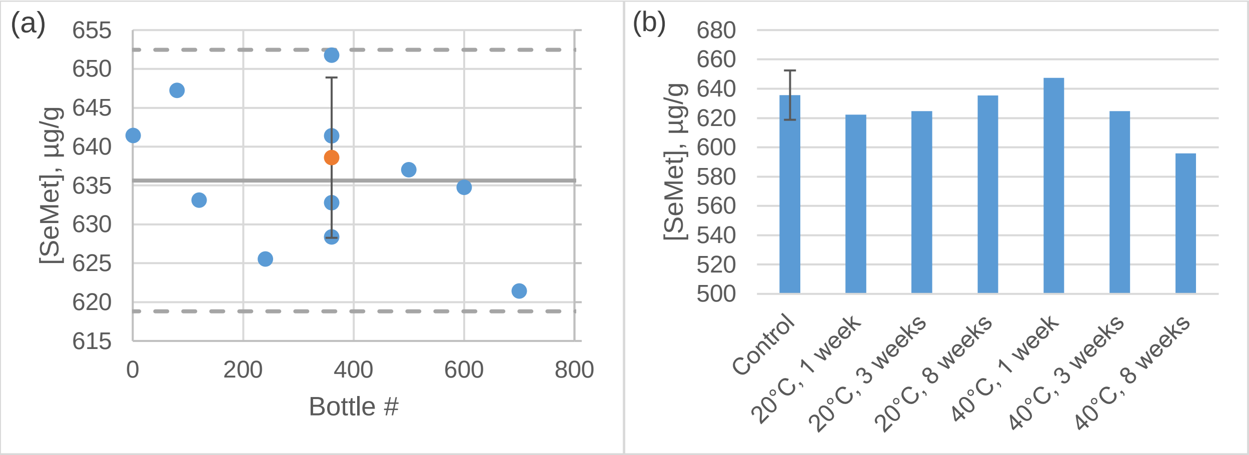

Figure S1: (a) Individual measured values for SeMet in SEEY-1 (blue points), including four replicate measurements from a single bottle (orange point), fall within the expanded uncertainty (dashed lines) of the certified value (solid line); (b) SeMet measurements for bottles subjected to isochronous stability test conditions, compared to the consensus value and uncertainty (k = 2) for all other measurements, show stability at all storage conditions, except 40°C for eight weeks where a 6% decrease in measured SeMet was observed

When results of individual measurements are plotted as a function of CRM bottle number (Figure S1a), no apparent trends were observed. Trends in this type of plot could indicate issues encountered during bottling which resulted in inhomogeneity; for example, progressive contamination, or the loss/gain of moisture in the dried yeast material.

Results from the isochronous stability study indicate that SeMet content in SEEY-1 does not change significantly when the bottle is stored in various temperature conditions, with the exception of 8 weeks at 40°C. Therefore, while it is recommended to store the CRM is the freezer (control conditions of approximately -20°C), temporary periods where the material is exposed to higher temperatures, such as during transport, will not degrade the analytes of interest.

Note that total Se measurements were performed for some of the isochronous stability samples: 20°C for 2 weeks, 40°C for 3 weeks, and 40°C for 8 weeks. The only measurement significantly different than the control was the sample held at 40°C for 8 weeks. This suggests that the decreased concentration of SeMet measured in this sample may partially be a result of a complete loss of the analyte rather than due to the conversion of SeMet to another Se species. Likely, it is actually a combination of both types of loss, as total Se only decreases by 2% after 8 weeks 40°C (compared to a 6% decrease in measured SeMet).

References for the Supplementary Information

1. Yang, L.; Sturgeon, R.E.; McSheehy, S.; Mester, Z. Comparison of extraction methods for quantitation of methionine and selenomethionine in yeast by species specific isotope dilution gas chromatography-mass spectrometry. *J. Chromatogr. A.* **2004,** *1055*, 177-184. DOI: 10.1016/j.chroma.2004.09.018
2. Yang, L.; Mester, Z.; Sturgeon, R.E. Determination of methionine and selenomethionine in yeast by species-specific isotope dilution GC/MS. *Anal. Chem.* **2004,** *76*, 5149-5156. DOI: 10.1021/ac049475p
3. Fietzke, J.; Liebetrau, V.; Günther, D.; Gürs, K.; Hametner, K.; Zumholz, K.; Hansteen, T.H.; Eisenhauer, A. An alternative data acquisition and evaluation strategy for improved isotope ratio precision using LA-MC-ICP-MS applied to stable and radiogenic strontium isotopes in carbonates. *J. Anal. At. Spectrom.* **2008,** *23*, 955-961. DOI: 10.1039/b717706b
4. LeBlanc, K.L.; Le, P.M.; Meija, J.; Ding, J.; Melanson, J.; Mester, Z. Preparation and certification of natural and ^{82}Se-labelled selenomethionine reference materials. *J. Anal. At. Spectrom.* **2021,** *36*, 416-428. DOI: 10.1039/D0JA00411A
5. National Institute of Standards and Technology. NIST Uncertainty Machine. https://uncertainty.nist.gov (accessed: 21 July 2022).
6. IUPAC. Commission on Isotopic Abundances and Atomic Weights. https://ciaaw.org/ (accessed: 21 July 2022).
7. National Institute of Standards and Technology. NIST Consensus Builder. https://consensus.nist.gov (accessed: 21 July 2022).
